# Clinical utility of procalcitonin in febrile infants younger than 3 months of age visiting a pediatric emergency room: a retrospective single-center study

**DOI:** 10.1186/s12887-021-02568-5

**Published:** 2021-03-04

**Authors:** Jun-Sung Park, Young-Hoon Byun, Jeong-Yong Lee, Jong Seung Lee, Jeong-Min Ryu, Seung Jun Choi

**Affiliations:** 1 Department of Pediatrics, Asan Medical Center, University of Ulsan College of Medicine, 88 Olympic-ro 43-gil, Songpa-gu, Seoul, 05505 Republic of Korea; 2 Department of Emergency Medicine, Asan Medical Center, University of Ulsan College of Medicine, Seoul, Republic of Korea

**Keywords:** Febrile infant, Neonate, Procalcitonin, Severe bacterial infection

## Abstract

**Background:**

Fever in infants under 90 days of age is highly likely to be caused by a severe bacterial infection (SBI) and it accounts for a large number of patients visiting the pediatric emergency room. In order to predict the bacterial infection and reduce unnecessary treatment, the classic classification system is based on white blood cell (WBC) count, urinalysis, and x-ray, and it is modified and applied at each center by incorporating recently studied biomarkers such as c-reactive protein (CRP) or procalcitonin (PCT). This study analyzed the usefulness of PCT in predicting SBI when applied along with the existing classification system, including CRP, among infants less than 90 days old who visited with a fever at a single institution pediatric emergency center.

**Methods:**

We retrospectively reviewed the medical records of patients younger than 3 months of age who presented with fever at the Seoul Asan Medical Center pediatric emergency room between July 2017 and October 2018.

**Results:**

A total of 317 patients were analyzed, and 61 were diagnosed with SBI, among which urinary tract infection (UTI) accounted for the largest proportion (55/61, 90.2%). There were differences in WBC, neutrophil proportion, CRP, and PCT between the SBI group and the non-SBI group, and the AUC values of WBC, CRP, and PCT were 0.651, 0.804, and 0.746, respectively. When using the cut-off values of CRP and PCTs as 2.0 mg/dL and 0.3 ng/mL, respectively, the sensitivity and specificity for SBI were 49.2/89.5, and 54.1/87.5, respectively. WBC, CRP, and PCT were statistically significant for predicting SBI in multivariate analysis (odds ratios 1.066, 1.377, and 1.291, respectively). When the subjects were classified using the existing classification criteria, WBC and CRP, the positive predictive value (PPV) and negative predictive value (NPV) were 29.3 and 88.7%, respectively, and when PCT was added, the PPV and NPV were 30.7 and 92%, respectively, both increased.

**Conclusion:**

PCT is useful for predicting SBI in children aged 3 months or less who visit the emergency room with a fever. It is useful as a single biomarker, and when used in conjunction with classic biomarkers, its diagnostic accuracy is further increased.

## Background

The most common cause of pediatric patients visiting the emergency room is fever [1]. Among them, especially those under 3 months, fever is often caused by a bacterial infection and it should be differentiated through examination [2]. According to some reports, the prevalence of severe bacterial infection (SBI) in infants under 3 months is only 10%, although in some developing countries, it is close to 30%, which places a heavy burden on the country [3–5]. The Rochester, Philadelphia, and Boston criteria have been widely used to evaluate the risk of a serious bacterial infection in infants less than 3 months of age with fever [6–9]. Although its use has been modified for each center, blood tests including white blood cell (WBC) count, urinalysis, and chest x-ray are routinely used. Also, various markers for predicting bacterial infection such as c-reactive protein (CRP) and procalcitonin (PCT) have been introduced [10–13].

Through this study, we confirmed the final diagnosis and prevalence of severe bacterial infection (SBI) in children under 3 months of age who visited the pediatric emergency medical center of a tertiary medical institution with very good medical accessibility and evaluated the effectiveness of the relevant clinical and laboratory parameters in the emergency room.

## Methods

### Study design, setting, participants

We retrospectively reviewed medical records of patients younger than 3 months of age who presented with fever at the Asan Medical Center pediatric emergency room between January 2017 to June 2018. This study was approved by the institutional review board of the Asan Medical Center, Seoul, Korea (IRB number: 2020–0850). All methods were performed in accordance with the relevant guidelines and regulations.

We excluded cases of being transferred to another hospital without examination, when full diagnostic testing was not performed for whatever reason (e.g., insufficient blood sampling), or temporary hyperthermia patients who had exceeded 28 days of age, were in a good general condition, their fever did not exceed 38 °C, and it did not recur after spontaneous resolution without antipyretics. When influenza was confirmed by a rapid antigen test after visiting during the influenza epidemic period, an antiviral agent was prescribed and the patient was discharged without any additional blood, urine, or x-ray tests being conducted. In the case of a revisit, cases undergoing a full evaluation were included, and if there were multiple revisits, the worst results were selected for inclusion.

We collected medical records and laboratory tests, including the present history, highest degree of fever, duration of fever, physical exams, vital signs, complete blood count (CBC), electrolytes, chemistry, CRP and PCT, and an x-ray. In addition, molecular biologic tests and cerebrospinal fluid (CSF) studies were performed as needed.

**Fig. 1:**
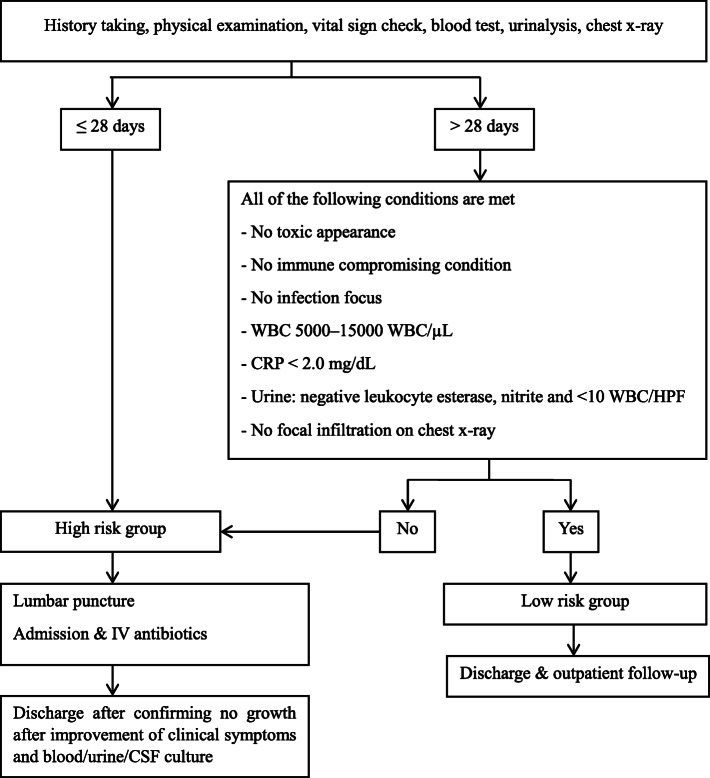
Diagnosis and treatment process for study patients

The diagnostic and treatment processes are shown in Fig. 1. Patients who exceeded 28 days of age with abnormal findings on the physical examination, x-ray, or laboratory test results were classified as high-risk. Patients under 28 days were classified into the high-risk group if they were treated with empirical antibiotics after lumbar puncture and hospitalization. If the age exceeded 28 days and they showed normal findings on the physical exam, x-ray, and laboratory tests, they were classified into the low-risk group and discharged without antibiotic treatment.

### Laboratory tests measurements

For the PCT test, an Enzyme-Linked Fluorescent Assay using an ADVIA Centaur® BRAHMS PCT assay (ADVIA Centaur XPT, Siemens) was used according to the instructions of the manufacturer. For the CRP test, a latex agglutination assay using CRPL3, Roche (Cobas 8000, Roche) was used according to the instructions of the manufacturer. WBC and absolute neutrophil count (ANC) were measured using an automated cell counter.

### Clinical diagnosis

SBI was defined as the isolation of bacterial pathogens from the blood, cerebrospinal fluid, or fecal samples, or urine cultures at 100,000 CFUs/mL. Non-bacterial infection was defined as a case in which a bacterial culture test was negative but another infection such as a viral origin was suspected based on the medical history, and the viral polymerase chain reaction (PCR) results obtained from each sample were considered. The diagnostic criteria for each system are as follows:

- Urinary tract infection (UTI): isolation of > 100,000 CFU/mL of a single organism [14]. The specimen was obtained by a urine collection bag.
- Pneumonia: positive sputum or pleural fluid culture or clinically lobar pneumonia on chest radiograph.
- Bacteremia: positive blood culture.
- Bacterial meningitis: positive CSF culture.
- Omphalitis: positive umbilicus pus culture with clinical symptoms of omphalitis (e.g., erythema, pus discharge, tenderness and swelling of the periumbilical area).
- Non-bacterial infection: negative bacterial culture for all specimens and the symptoms were completely resolved without treatment or did not show a relapse when antibiotics were stopped.
- Non-bacterial upper respiratory tract infection (URTI): upper respiratory infection symptoms including rhinorrhea, cough, and sputum without evidence of a lower respiratory infection (LRTI) or bacterial pneumonia.
- Non-bacterial LRTI: pneumonic infiltration on chest radiograph or abnormal lung sounds with wheezing or crackles without evidence of bacterial pneumonia.
- Non-bacterial meningitis: CSF pleocytosis > 20 /μL for infants ≤28 days of age, > 10 /μL for infants > 28 days of age who were negative for bacterial culture [15].
- Influenza: positive for influenza PCR or influenza rapid antigen test.
- Viral AGE: positive for stool viral PCR, including rotavirus, norovirus, astrovirus, sapovirus and enterovirus and negative for stool bacterial culture.
- Kawasaki disease: fever of 5 days or more and 4 or more of the following 5 symptoms are present: conjunctival injection, cervical lymphadenitis, strawberry tongue, skin rash, palmar erythema [16].
- Fever without focus: no localized symptoms, no pathogen is detected in any of the samples, and the symptoms are completely resolved without treatment or did not show a relapse when antibiotics were stopped.

### Statistical analysis

The general clinical conditions and demographics were described. Patients were categorized into groups with and without a bacterial infection. For a comparison of the clinical data between the two groups, a Mann-Whitney u-test was performed for continuous variables that did not follow a normal distribution, and the χ-square test was performed for categorical variables. The diagnostic utility of markers considered to detect SBI was investigated by using receiver-operating characteristic (ROC) curves and a regression model. The cut-off values for evaluating the accuracy of WBC and CRP were 15,000/μL and 2.0 mg/dL, respectively, which are used in research hospitals. *P*-value < 0.05 was considered statistically significant. Analyses were performed with PASW Statistics Version 18.

## Results

**Fig. 2:**
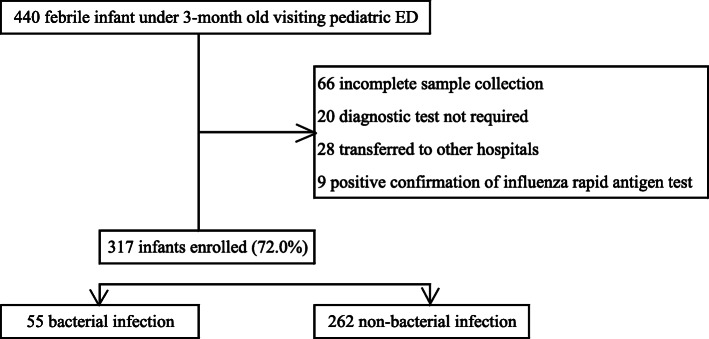
Study population description

A total of 440 infants under 3 months of age visited the pediatric emergency center during the study period. Among them, 66 patients with incomplete laboratory tests on any of the PCT, WBC, UA, or x-ray, 20 patients with transient hyperthermia, 28 patients who had been transferred to other hospitals, and 9 patients who did not undergo other tests due to a positive influenza antigen test were excluded. Thus, a total of 317 patients were enrolled in this study (Fig. 2). Among them, 61 patients were diagnosed with an SBI, and UTI was the most common, affecting 55 patients (Table 1).

**Table 1:** Final diagnosis of patients (*n* = 317)

	n (%)
Bacterial infection (*n* = 61)	
UTI	55 (90.2)
Pneumonia	3 (5.0)
Bacteremia	1 (1.6)
Bacterial meningitis	1 (1.6)
Omphalitis	1 (1.6)
Non-bacterial infection (*n* = 256)	
Non-bacterial URTI	28 (10.9)
Non-bacterial LRTI	11 (4.3)
Non-bacterial meningitis	8 (3.1)
Influenza	6 (2.3)
Viral AGE	3 (1.2)
Kawasaki disease	1 (0.4)
Fever without focus	199 (77.8)

Data presented as number (percentage)

*Abbreviation*: *AGE* acute gastroenteritis, *LRTI* lower respiratory tract infection, *URTI* upper respiratory tract infection, *UTI* urinary tract infection

**Fig. 3:**
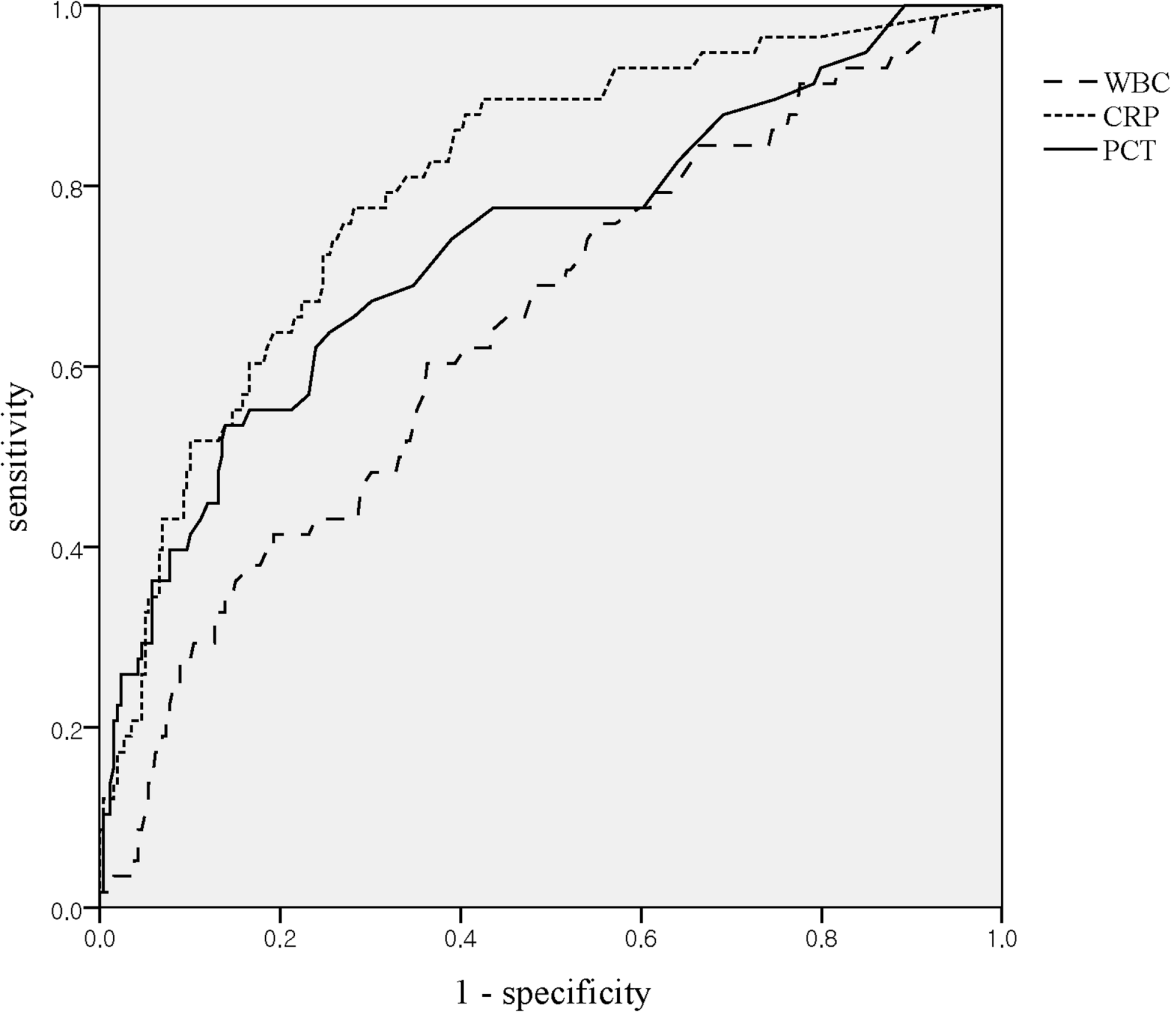
ROC curve analysis to determine the associations between the parameters and SBI

When comparing the SBI and non-SBI, there were more boys in the SBI and the WBC, neutrophil proportion, CRP, and PCT were higher in the SBI group (Table 2). There were no statistically significant differences in age, weight, duration of fever, peak temperature, or vital signs between the two groups. In the ROC curve analysis performed to confirm the association of WBC, CRP, and PCT with SBI, the areas under the curve (AUC) were 0.651, 0.804, and 0.746, respectively (*p*-values < 0.001, < 0.001, < 0.001, respectively) (Fig. 3). The cut-off value of PCT was set as 0.3 ng/mL, derived from the maximum value of Youden’s index using the ROC curve analysis, where it had the most similar sensitivity/specificity to the existing classification criteria, and CRP ≥2.0 mg/dL (CRP ≥2.0 mg/dL, 49.2/89.8; PCT ≥0.3 ng/mL, 54.1/87.5). When using cut-off values of WBC, CRP, and PCTs as 15,000/μL, 2.0 mg/dL and 0.3 ng/mL, respectively, the sensitivity and specificity for detecting SBI were 45.9/69.1, 49.2/89.5, and 54.1/87.5, respectively (Table 3).

**Table 2:** Patient characteristics and clinical data

Characteristic	Bacterial infection (*n* = 61)	Non-bacterial infection (*n* = 256)	*P* value
Demographics			
Sex (male)	46 (75.4)	148 (57.8)	0.013
Age (days)	32.0 (20.0–67.0)	30.0 (20.0–63.8)	0.627
Body weight (kg)	5.6 (5.0–6.2)	5.4 (4.7–6.1)	0.209
Fever onset (h)	3 (1–7)	4 (2–10)	0.930
Vital sign			
Body temperature (°C)	38.4 (38.1–38.8)	38.2 (38.0–38.5)	0.388
SBP (mmHg)^a^	92.0 (89.0–106.0)	89.0 (80.0–101.0)	0.132
DBP (mmHg)^a^	61.0 (54.0–68.0)	55.0 (48.0–65.5)	0.064
Heart rate (/min)^a^	178.0 (166.0–189.0)	173.0 (158.0–190.0)	0.209
Respiratory rate (/min)	44.0 (40.0–49.5)	46.0 (40.0–50.0)	0.418
Laboratory test			
Hb (g/dL)	10.4 (9.8–10.9)	10.6 (9.9–11.4)	0.063
WBC (/uL)^a^	13.4 (10.3–18.6)	11.0 (7.6–14.5)	< 0.001
Neutrophil proportion (%)	51.6 (41.6–59.0)	39.5 (30.4–50.0)	< 0.001
CRP (mg/dL)	1.9 (1.1–3.8)	0.4 (0.1–1.2)	< 0.001
PCT (ng/mL)	0.3 (0.2–1.0)	0.2 (0.1–0.2)	< 0.001

Results are presented as median (interquartile range) or number (%)

^a^Results are presented as mean (95% confidence interval)

**Table 3:** The predictivity of the laboratory parameters for severe bacterial infection

	Sensitivity	Specificity	PPV	NPV
WBC (≤5000 or ≥ 15,000/μL)	45.9	69.1	26.2	84.3
CRP (≥2.0 mg/dL)	49.2	89.5	52.6	88.1
Procalcitonin (≥0.3 ng/mL)	54.1	87.5	50.8	88.9

*Abbreviations*: *WBC* white blood cell, *CRP* C-reactive protein, *PPV* positive predictive value, *NPV* negative predictive value

When performed with variables that were statistically significant in the univariate analysis, only WBC, CRP, and PCT were statistically significant in the multivariate analysis (odds ratios 1.066, 1.377, 1.291, respectively; *p*-values 0.035, < 0.001, 0.03, respectively) (Table 4). Assuming that the patients included in the study were classified using only two variables, the CRP and WBC, the sensitivity, specificity, positive predictive value (PPV), and negative predictive value (NPV) for predicting SBI were 67.2, 61.3, 29.3, and 88.7, respectively. (Table 5). When PCT was added to WBC and CRP in the same manner, the sensitivity, specificity, PPV, and NPV in predicting SBI were 80.3, 55.5, 30.1, and 92.2, respectively.

**Table 4:** Multivariate logistic regression analysis for the prediction of severe bacterial infection

	Odds ratio	95% confidence interval	*P* value
Sex	1.697	0.843–3.417	0.139
WBC	1.066	1.005–1.132	0.035
Neutrophil proportion	1.003	0.988–1.018	0.701
CRP	1.377	1.155–1.642	< 0.001
Procalcitonin	1.291	1.025–1.627	0.030

*Abbreviations*: *WBC* white blood cell, *CRP* C-reactive protein

**Table 5:** Predictivity for diagnosing severe bacterial infection using the test results

	Total			
	sensitivity	specificity	PPV	NPV
WBC (< 5000 or > 15,000 /μL) & CRP (≥2.0 mg/dL)	67.2	61.3	29.3	88.7
WBC (< 5000 or > 15,000 /μL) & CRP (≥2.0 mg/dL) & Procalcitonin (≥0.3 ng/mL)	80.3	55.5	30.1	92.2

*Abbreviations*: *SBI* severe bacterial infection, *WBC* white blood cell, *CRP* c-reactive protein

## Discussion

In this study, it was confirmed that PCT, as well as WBC and CRP, which are classic biomarkers, are useful in assessing the risk of SBI in infants aged 3 months or less who visit the emergency room due to fever. To date, the Rochester, Philadelphia, and Boston criteria are frequently referenced to evaluate the risk of bacterial infection in infants under 3 months of age with fever [6–9]. Among these criteria, WBC and urinalysis are common evaluation criteria along with blood tests, chest x-ray, CSF, stool tests, etc. that are selectively used. In the pediatric emergency room unit of this research hospital, these criteria have been modified to determine the disposition of infants less than 3 months of age with fever (Fig. 1). This study is meaningful in that it evaluated the usefulness of PCT, which is not included in the classic three criteria or the research hospital protocol, and it confirmed that PCT is useful for predicting SBI with or without the addition of conventional laboratory tests in children less than 3 months old with fever.

WBC, which is used as a traditional biomarker, was found in this study to be inferior in diagnostic accuracy when used alone (sensitivity 45.9, specificity 69.1, PPV 26.2, NPV 85.3, AUC 0.651). However, it was found to be a meaningful variable in multivariate analysis (OR 1.066, CI 1.005–1.132, *P* = 0.035), and recent studies reported that WBC is a useful variable, so we cannot conclusively say that WBC is not a useful biomarker based on the results of this study alone [17]. Therefore, it is necessary to carefully study whether WBC can be used as an effective biomarker or be replaced by newly developed biomarkers.

In this study, there were few SBIs except UTIs among patients enrolled over a period of 18 months. The epidemiology of bacterial infection in infants is changing worldwide. In particular, invasive bacterial infections are decreasing and the rate of UTIs is increasing [18, 19]. In the most recent study, the majority of SBIs were identified as UTIs [20]. It was also confirmed that more than 30% of invasive bacterial infections were accompanied by a UTI [21]. This study was conducted on a patient group, the majority of which had a UTI, so it did not deviate from the global trend. Nevertheless, since it is a study involving a small number of patients with IBI and non-UTI SBI, caution is required when applied to various diagnostic spectrums, and thus additional large-scale studies should be conducted.

However, caution is needed in the interpretation of the results since the urine collection method used in this study applied a urine collection bag instead of suprapubic aspiration or transurethral catheterization. In the most recent studies, it has been confirmed that a urine collection bag is primarily used in the pediatric ED to reduce patient pain and complications associated with the collection procedure [22]. In this study, in order to minimize contamination that may occur during urine collection using a collection bag, 2% chlorhexidine was used to sterilize the urethral opening three times or more by drawing a concentric circle, and after it had completely dried, the collection bag was attached. During the sterilization, traction of the prepuce was performed for boys, and for girls, the wrinkles of the labium were spread. When urinating with feces passage, it was regarded as contaminated, so it was collected again. If even a little urine leaked out, it was considered exposed to the external contamination and collected again. Only specimens identified above 100,000/CFU with a single organism in the urine culture were reported as positive. Although a UTI was defined only based on the bacterial culture results, there were no UTI patients without pyuria. Although being aware of the limitations of the urine collection bag method, it was chosen in order to avoid performing an invasive procedure, and all of the UTIs were accompanied by pyuria, minimizing false positives. In order to interpret the results accurately in additional research, it is necessary to actively use transurethral catheterization or suprapubic aspiration [23].

In this study, when PCT was applied together with WBC and CRP, the sensitivity, PPV, and NPV increased from 67.2, 29.3, and 88.7 to 80.3, 30.1, and 92.2, respectively. This may not seem very dramatic, and it may be interpreted as not being cost-effective as it increases unnecessary hospitalization. However, several studies in adults have already reported that the use of PCT is rather cost-effective to predict SBI [24–26]. Bacterial infection in infants can have serious consequences if adequate treatment is not provided. Nathan et al. prioritized the sensitivity of the prediction rule by specifying a relative cost of 100 to 1 for failure to identify an SBI vs incorrectly predicting an SBI [17]. According to Carrie et al., although more hospitalization, lumbar puncture, and antibiotic treatment were performed while completing more diagnostic tests using augmented criteria, advanced viral PCR technology and changes in discharge criteria resulted in a reduced overall cost and length of stay, and a reduction in the unnecessary antibiotics duration [4]. Therefore, it might be better to use PCT as a biomarker to minimize misclassification and to find a desirable combination of biomarkers and cut-off values through prospective research to reduce the total cost. Furthermore, performing tests for biomarkers sequentially according to algorithms with cut-off values and minimizing unnecessary tests, rather than testing for all eligible biomarkers at the same time as in current strategies, may be a good approach and needs further study.

The median (IQR) fever duration of the patients in this study was 3 (1.5–9.0) hours, which was shorter than that of a similar study [27, 28]. According to previous studies, CRP and PCT do not detect SBI well at the very beginning of the infection, so their sensitivity and negative predictive value may decrease [29, 30]. This study showed sufficient diagnostic accuracy to discriminate SBI even in patients who visited the hospital after a median duration of 3 h, so it could be confirmed that CRP and PCT are useful in predicting SBI even in areas with high medical accessibility. This result may act as a bias for applications with a longer fever duration. However, considering that the peak level of each biomarker is maintained for more than 24 h, it will not act as a serious bias. In fact, Karen et al., Olaciregui et al., and Borja et al. also reported the effectiveness of PCT regardless of fever onset duration [10, 13, 31]. Therefore, even in cases with a longer febrile illness duration than in this study, it is considered that there might be no major bias in applying our results.

There were more boys in the SBI group in this study. This is thought to be because most of the SBIs were UTIs, and among infants, males are more susceptible to UTIs [32]. However, sex was not statistically significant in multivariate logistic regression analysis in predicting an SBI.

The limitations of this study are that the total number of enrolled patients was only 317 and that it was a retrospective study. Patients evaluated as transient hyperthermia returned home without examination in consideration of avoiding unnecessary pain, radiation exposure, and length of stay, and were excluded from this study. Although revisiting patients are included in the study and SBIs are hard to be missed, it is possible that patients with mild infections may have been missed and the results of the study might be overestimated. In addition, although patients excluded due to incomplete examination or being transferred to other hospital were not evaluated for their disease severity, the possibility of selection bias cannot be excluded. In order to minimize those biases, body temperature was measured repeatedly during the ED stay to discriminate fever from transient hyperthermia, and re-sampling was conducted for incomplete sampling if the parental opposition was not severe. Also, most of the patients had UTIs. Given the relatively low prevalence of bacteremia, bacterial central nerve system (CNS) infections, and bacterial pneumonia, it is necessary to include a wider variety of disease entities through larger studies. In addition, it is necessary to conduct a prospective study that actually classifies patients using the new classification system to predict the risk of SBI. Good criteria to more effectively classify patients aged less than 3 months with fever, which consume a lot of medical care, need to be established.

## Conclusion

PCT is useful for predicting SBI in children aged 3 months or less who visit the emergency room with a fever. It is useful as a single biomarker and it is even better when combined with traditional biomarkers (WBC and CRP) to improve their diagnostic accuracy.

## Data Availability

The datasets generated and analyzed during the current study are not publicly available due that institutional review board of the Asan Medical Center, Seoul, Korea (IRB number: 2020–0850) did not allow to share with out-of-hospital facility because of ethical consideration but are available from the corresponding author on reasonable request after permission of the institutional review board and de-identification of data.

## Abbreviations

WBC: White blood cell
CRP: C-reactive protein
PCT: Procalcitonin
SBI: Severe bacterial infection
PPV: Positive predictive value
NPV: Negative predictive value
CBC: Complete blood count
CSF: Cerebrospinal fluid
ANC: Absolute neutrophil count
PCR: Polymerase chain reaction
UTI: Urinary tract infection
URTI: Upper respiratory tract infection
LRTI: Lower respiratory infection
ROC: Receiver-operating characteristic
AUC: Area under the curve
IQR: Interquartile range
CNS: Central nerve system
